# Factors Related to Client Satisfaction with Community Based Health Insurance Services During COVID-19 Pandemic in Central Uganda: A Mixed Methods Healthcare Facility Based Study

**DOI:** 10.24248/eahrj.v8i2.785

**Published:** 2024-06-26

**Authors:** Stevens Kisaka, Frank K. Tumwebaze, Simon Kasasa

**Affiliations:** a Makerere University School of Public Health, Uganda; b Business School, Eastern and Southern African Management Institute (ESAMI), Arusha, Tanzania

**Keywords:** Client satisfaction, community-based health insurance, COVID-19, Uganda

## Abstract

**Background:**

During the COVID-19 lockdowns, healthcare services were disrupted and community-based health insurance (CBHI) schemes could not operate efficiently. This study assessed the level of client satisfaction with CBHI schemes, associated factors, and service provider perspectives in central Uganda.

**Methods:**

This was an explanatory sequential mixed-methods (quantitative - qualitative) study that was conducted between March and September 2021. In the first phase, a cross-sectional study among the 365 clients of the CBHI schemes who were aged ≥18 years old. The participants were recruited consecutively as they reported to the healthcare facility. Quantitative data were collected at patient exit using a piloted semi-structured interviewer-administered questionnaire. In the second phase, qualitative data were collected through 11 key informant interviews. These data were analyzed using a deductive thematic analysis approach. Modified Poisson regression was used to assess factors associated with client satisfaction and a p-value ≤0.05 at a 95% confidence interval was considered to be statistically significant.

**Findings:**

Of the total number of participants, 38.9% (142/365) were “satisfied” with the CBHI services. Less satisfaction was associated with secondary level of education or above (adjPR = 0.55, 95% CI: 0.36–0.85, *P=.007*); residing beyond 16 kilometers from the healthcare facility (adjPR = 0.68, 95% CI: 0.41–0.95, *P=.014*); staying on the scheme for over 3 years (adjPR = 0.71, 95% CI: 0.51–0.99, *P=.046*); and good knowledge about the CBHI (adjPR = 0.76, 95% CI: 0.58–0.99, *P=.040*). Irregular availability of healthcare workers and long waiting time affected client satisfaction.

**Conclusions:**

Satisfaction was considerably low during the lockdown. Lockdowns due to pandemics interrupt healthcare services and subsequently affect the satisfaction of CBHI clients with scheme services. Scheme managers need to identify facilities that are closer to enrolees and invest in technologies that reduce waiting time in the healthcare facility.

## BACKGROUND

To achieve universal health coverage, the 58th World Health Assembly (WHA) decided that countries must ensure that health financing incorporates a method for prepayment. This was borne out of the fact that fees are a key factor behind the poor utilization of healthcare services.^1^ Direct out of pocket (OOP) health payments remain a key barrier for access to healthcare services, hence health-related poverty. Therefore, the WHA resolution intended to not only enhance risk-sharing and reduce catastrophic health expenditure but also to prevent healthcare seekers from impoverishment.^2^

Countries in sub-Saharan Africa are trying to implement the WHA resolution through policies that seek to accelerate utilization of medical insurance with involvement at the community level. This aims to promote equitable use of healthcare services.^3^ In Uganda, the Ministry of Health encourages people to join health insurance schemes, one of which is the type referred to as “community-based health insurance” (CBHI). In 1995, Government introduced several community healthcare-financing projects to allow for optional strategies to healthcare financing.^4^ Subsequently, CBHI schemes are sprouting, and they are characterized by voluntary membership, nonprofit objectives, linkages with a healthcare provider, pooling risk, joint aid trust, enrollment, and unity at the community level.^5^

About 30 CBHI are operating in different parts of Uganda and serve about 155,057 people.^6^ Notably, in areas where they operate, these schemes cover approximately 10% of the population they target.^7^ The low coverage is worsened by the high dropout rates that characterize such schemes.^8^ Much as client satisfaction is a major driver of consumer retention and enrollment in health insurance,^9^ little is known about it as regards CBHI schemes. If the levels of customer satisfaction are not assessed, then the CBHI schemes are likely not to grow, and rural populations will keep being driven into household poverty due to medical bills.

Studies conducted elsewhere have attributed client satisfaction with CBHI to socio-demographic characteristics.^5,10^ Other factors associated with satisfaction include client perception of laboratory services, the healthcare providers' friendliness, knowledge about the insurance, the availability of human resources, and access to medicines.^10-12^ The few studies that have been conducted about CBHI in Uganda have only assessed enrolment,^4^ policy makers' and health managers' views on CBHI,^13^ willingness to pay,^7^ utilization,^14^ characteristics,^15^ and lessons from previous CBHI efforts.^16^

During the COVID-19 season in Uganda, which commenced in March 2020, stringent measures to enable social distancing were implemented. These comprised lockdown measures consisting of curfews, travel restrictions, the closure of various institutions, bans on public gatherings, and border closures.^17^ These restrictions resulted in various negative effects related to healthcare service delivery, as some patients could not even access health facilities.^18^ However, the issue of CBHI client satisfaction during such periods has been largely ignored.

Insufficient information on client satisfaction and provider experiences during such pandemic-related restrictions means this information isn't available to be used to improve the performance of CBHI schemes. Consequently, client experiences cannot be harnessed to drive better service delivery, expansion and retention in the schemes. Therefore, this study sought to determine the level of satisfaction and associated factors among CBHI clients during the 2020 COVID-19 lockdown. In addition, the study explored perceptions of scheme managers as well as healthcare service providers on client satisfaction. Information generated may guide the development and improvement of CBHI programs in the country, even during similar lockdowns.

## METHODS AND MATERIALS

### Study Setting

The study was conducted in healthcare facilities that served community health insurance schemes in Luwero, Nakasongola, and Nakaseke districts, in central Uganda. The schemes were started in 1999 and currently cover 21,504 beneficiaries from 319 families. These are served by 2 hospitals and 7 lower-level healthcare facilities. These schemes were started by non-governmental organizations (NGO) during the time the beneficiary communities were recovering from the effects of the 1980s civil war. There are some minor disparities in benefit packages, premiums, co-payments, and other operational details between the schemes. Generally, each individual pays a premium of around USD 2 per year, though co-payment plans vary for each sub-scheme. The hospital offers a 12% discount on the hospital bill to scheme members.^6^

### Study Design

This was an explanatory sequential (quantitative-qualitative) mixed methods study. Data were collected sequentially, and in the first phase, a cross-sectional study was conducted between March and September 2021 among the clients of the CBHI schemes to collect quantitative data on the level of satisfaction with the scheme as well as factors associated with it. In the second phase, key informant interviews were used to collect qualitative data from CBHI scheme managers as well as healthcare providers.

### Study Population

The study included adult (≥18 years old) CBHI enrollees who had reported to the healthcare facility to use the CBHI services. In addition, scheme managers and healthcare providers were included. The CBHI clients who were too sick and therefore not capable of consenting to offer responses were excluded from the study.

### Sample Size and Sampling

For quantitative data, the sample size was calculated using the single population proportion formula for cross-sectional studies while assuming client satisfaction level of 42%,^12^ a 5% margin of error at a 95% confidence interval. After adjusting for a predicted 10% non-response rate, a final sample size of 365 respondents was calculated. For qualitative data, managers of CBHI schemes and healthcare providers were interviewed consecutively until saturation was realized.

Health facilities were purposively selected based on provision of service to the CBHI scheme clients. The sampling unit was a patient who had sought care under the CBHI scheme. All the patients were enrolled consecutively. A structured questionnaire, developed based on the literature review, was used to record patient socio-demographics as well as their satisfaction and various explanatory factors.

For qualitative data, after interviewing 11 of the scheme managers and healthcare providers, saturation was realized, and the interviews were stopped. The point of data saturation was determined to have been reached when no new or relevant information materialized from the additional interviews conducted as earlier described by various authors.^19^

### Data Collection Procedures

Data were collected using a semi-structured questionnaire and a key informant interview guide. The questionnaire on client satisfaction and potential explanatory variables was administered by the interviewer. All data collection tools had been pretested and were in English and Luganda since the population generally speaks one of the two languages. The study tools were pre-tested on 20 members of a CBHI scheme in Bushenyi district, in the western part of Uganda. The purpose of this was to detect discrepancies in the tools; identify the difficulties encountered by the respondents; recognize the possible misunderstood and un-understood aspects of the tool; as well as other challenges related to the study tools. Appropriate steps were taken to rectify the problematic areas of the questionnaires, depending on the challenge recognised.

Obtaining administrative permission and informed consent was the first step in the data collection process. The interviews were conducted in a private environment after obtaining informed consent. The interview proceeded according to the interview tool. For key informant interviews, the interviews were digitally audio-recorded using an audio recorder device (SONY ICD PX333 Digital Voice Recorder®) in addition to notes that were taken on major points. Upon ending the interview, the interviewer orally thanked the participant in the most appropriate language and way possible.

### Data Processing

Prior to ending the interview process, the data collector checked the questionnaires to ensure that all the sections had been completed. For each participant, the completed interview questionnaire was doubly entered into Epi Info™ version 7.2.2.6 statistical software (CDC, February 2, 2018), while checking for any errors in the values or responses to the variables. Data analysis was conducted using Stata 16 software (StataCorp, June 2019). For qualitative data, independent data analysts transcribed the data into written text. NVivo 11.4.1® software (QSR International, 2017) was used to organize this data for analysis.

### Client's Overall Satisfaction

The independent variable was clients' overall satisfaction with the CBHI scheme. The study used a total of nine items that were associated with satisfaction. These nine items included: 1) trust by clients in the scheme management; 2) opening hours of the scheme; 3) process of collecting insurance cards; 4) time period / interval between registration and use of the insurance; 5) schedule of premium payment; 6) information provided by scheme; 7) scheme packages (range of services); 8) commitment to stay on scheme and; 9) potential to recommend the schemes to expand to other areas. Each of these items had a five-point Likert scale that was designed to range from strongly disagree (0 points), disagree (1 point), not decided (2 points), agree (3 points), and strongly agree (4 points). These items in the data tool were subjected to a reliability test. The internal consistency of these ten items was calculated, and a Cronbach's alpha above 0.701 was generated. Previous authors have determined a Cronbach α of 0.70 as acceptable.^20^ A respondent was labeled as “satisfied” if their response was higher than or equivalent to the median score for satisfaction questions. If not, then the respondent was labeled as “not satisfied.” The same approach has been used in earlier studies about satisfaction with health insurance in Ethiopia.^10^

### Knowledge of Respondents on CBHI Benefit Packages

To measure knowledge, respondents were asked seven items or questions that are related to the benefits of the CBHI scheme. These questions included: a) CBHI is a good way of assisting clients with health expenditure; b) CBHI covers people for care only from public health institutions; c) CBHI covers patients for care only within the country; d) CBHI doesn't cover expenses on patient transportation; e) CBHI covers care for outpatients only; f) CBHI covers care only for inpatients; and g) CBHI doesn't cover medical care for cosmetic purposes. Respondents were considered to have “adequate knowledge” if they correctly answered more than five items as described above. Otherwise, the respondents were categorized as “not having adequate knowledge.”

### Socioeconomic Status (SES) of Respondents

Socioeconomic status was measured using a principal component analysis (PCA) based on possession of these items (yes/no): Radio, television, cell-phone, bicycle, motorcycle, motor vehicle, a piece of land, large farm animals (like cattle, goats, and sheep), small farm animals like poultry, and a manufactured bed. Included was also the nature of the walls of the house (no bricks/unburnt bricks/burnt bricks with mud/burnt bricks/stones with cement/other and specify). The principal component on which most assets loaded was used to generate an SES score for every participant. Participants were then grouped into SES tertiles, namely “lowest,” “middle” and “upper” (richest), in ascending order. This technique has been widely used to classify people according to socioeconomic rankings in surveys.^21^

### Levels of Healthcare Facilities From Which Medical Care Was Sought

To understand the levels of health care facilities in Uganda, there is a need to define the lowest unit, i.e., health center II. Such a facility serves a few thousand people, but the principle is that it must be able to handle simple and common conditions and diseases such as diarrhea. As a standard, this healthcare facility is led by an enrolled nurse who may work as a midwife. Other staff include at least 2 nursing assistants as well as a health assistant. At this level, only outpatient clinics are run, with key needs such as antenatal care, also addressed. The next level is the health center III, and every sub-country in Uganda must have a health center III. By policy, this level of healthcare facility must have at least 18 healthcare workers who are headed by a senior clinical officer. They are equipped with laboratories and have a maternity ward. However, they run general outpatient clinics without admission. Health center IV facilities operate like small hospitals in addition to the services that are found at level III facilities. They have wards to admit patients, and they are headed by senior medical officers. They are equipped to undertake emergence operations. By policy, the hospitals have all the amenities of a health center IV. However, hospitals have specialised clinics, for example, paediatrics, mental health, and others.

### Data Analysis.

### Quantitative data

At Univariate analysis, descriptive statistics were computed, for example, frequencies, medians (together with the interquartile range), as well as proportions and percentages. Bivariate analysis was performed to assess the relationship between each independent variable and client satisfaction as the outcome. Prevalence ratios were computed using a generalized linear model (GLM) analysis with the Poisson family and a log link with robust standard errors. The independent variables that had a *P* value <.20 at bivariable analysis and whose association with the outcome is scientifically plausible were included in the multivariable model. The best-fitting model was constructed following the logical model-building technique. Variables that were found to have a *P* value ≤.05 at a 95% confidence interval (95% CIs) were considered to be statistically associated with satisfaction.

### Qualitative Data

Multiple readers (two) reviewed the transcripts and identified the information that was related to client satisfaction. Under each theme, the information was deductively coded into sub-themes, and then patterns were identified to form the explanatory points of what is being observed. Key statements corresponding to the themes were presented together with quantitative findings as a way of augmenting the latter. In short, the purpose of the qualitative data was to help explain what had been observed in the quantitative data.

### Ethical Considerations

This study protocol, including the consent forms, were subjected to approval by the ESAMI Research Ethics Review Committee (ESAMI/DAT/40E/2021/02). The investigators ensured that participants were given full and adequate oral and written information about the nature, purpose, possible risks, and benefits of the study. They were given adequate opportunity to ask questions and allowed time to consider the information provided on a voluntary basis. The subject's signed and dated informed consent was obtained before conducting this study. Participants in this database were identified by their unique enrolment numbers. Only the PI had access to the participant identification list, which included their unique codes, full names, and the latest known address. All methods in this study were performed in accordance with the relevant guidelines and regulations.

## RESULTS

### Background Characteristics of the Respondents

The total number of study participants enrolled in this study was 365, reflecting a response rate of 100%. Of these, 223 (61.1%) were female, and the median (IQR) age for the entire sample was 42 (IQR: 21) years. The median distance from the healthcare facilities was 7 km (IQR: 46 km). Most of the respondents (167/365, 45.8%) were educated up to the primary level. In terms of employment, most of the respondents had something they were doing for purposes of income (257/365, 70.4%), and of these, 150/257 (58.4%) were farmers. Further, most of the respondents (213/365, 58.4%) were in the lowest tertile of socio-economic status. A larger proportion of respondents (233/365, 63.8%) had made the decision to enroll in the CBHI schemes by themselves, and the commonest reason for joining was cited as having heard about the good experiences of those that were on the CBHI schemes before (216/365, 59.3%). The rest of the background characteristics of the respondents are shown in Table 1.

**Table 1.** Respondents' Characteristics

Variables	Frequency (N = 365)	Percentage (%)
Age		
≤25	44	12.1
26-35	83	22.7
36-45	83	22.7
46 - 55	74	20.3
≥56	81	22.2
Sex		
Male	142	38.9
Female	223	61.1
Highest education level attained		
No formal education	76	20.8
Primary	167	45.8
Secondary	104	28.5
Post-secondary education	18	5.0
Religion		
Christian	300	82.2
Muslim	65	17.8
Marital status		
Single never married	26	7.1
Single divorced / widowed	73	20.0
Married	262	71.8
Family size		
1-4	42	11.5
5 – 8	223	61.1
≥ 9	100	27.4
Engaged in income-generating activity		
Yes	257	70.4
No	108	29.6
Occupation		
Farmer	150	58.4
Transport industry	30	11.7
Business	63	24.5
Professional	14	5.4
Socioeconomic status (SES)		
Lowest tertile	213	58.4
Middle tertile	98	26.9
Upper tertile	54	14.8
Knowledge		
Less knowledge	94	25.7
More knowledge	271	74.3
Person that made decision to join CBHI		
Respondent (him/herself)	233	63.8
Spouse of respondent	63	17.3
Other	69	18.9
Reasons for joining scheme		
Heard about good experience	216	59.3
Offers affordable care	70	19.2
I fall sick regularly	71	19.5
Other	8	1.9
Level of healthcare facility		
Private clinic	26	7.1
Level III	104	28.5
Level IV	28	7.7
Hospital	207	56.7

**Figure 1.**
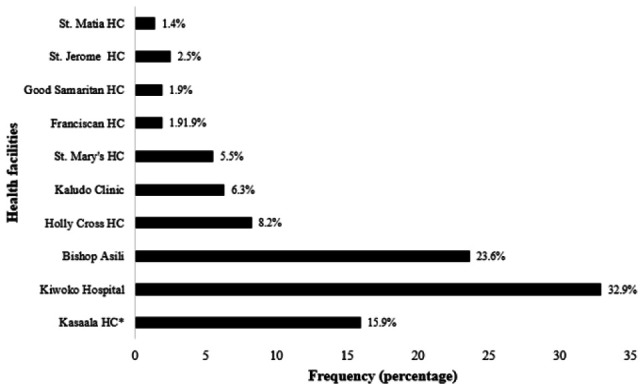
Distribution of Respondents According To Healthcare Facilities to Which Care Was Sought

In terms of specific healthcare facilities, Kiwoko and Bishop Asili hospitals had the most respondents in the sample, at 120/168 (32.9%) and 86 (23.6%), respectively. However, St. Jerome and St. Matia Mulumba, both healthcare center level III facilities, had the fewest respondents in the sample, at 9 (2.5%) and 5 (1.4%), respectively. The distribution of the respondents among the healthcare facilities included in this study is shown in Figure 1.

### Experiences of Respondents on CBHI Schemes

The respondents described their experience on the various sub-schemes, and 162/365 (44.4%) of them had been on the scheme for more than 3 years, while 72/365 (19.5%) had spent just one year on the CBHI. Those who had paid more than seven times since joining the scheme were 130 (35.6%), while 121 (33.1%) had paid less than three times by the time they were interviewed. In the previous year, 60/365 (16.4%) of the respondents had visited the healthcare facilities more than thrice, while 147/365 (40.3%) had visited only hospitals, compared to 118/365 (32.3%) who had visited only health center IIIs. The details of the experiences of the respondents are shown in Table 2.

**Table 2.** Experiences of Study Participants on Community Based Health Insurance Schemes in Central Uganda

Variables	Frequency (N = 365)	Percentage (%)
Length of enrolment		
≤1 year	72	19.5
>1 – 3 years	132	36.2
>3 years	162	44.4
Times paid so far		
≤3	121	33.1
4 - 6	114	31.2
>7 times	130	35.6
Health facility visited		
Only health center	118	32.3
Only hospital	147	40.3
Both	100	27.4
Frequency of health facility visiting		
1-3 times	305	83.6
>3 times	60	16.4
Happy with the chosen facilities		
Yes	286	78.6
No	79	21.4
Got prescribed drugs		
Yes	275	75.3
No	90	24.7
Received laboratory services as requested		
Yes	321	88.2
No	43	11.8

### Knowledge of Respondents on CBHI Benefit Packages

About a quarter of the respondents, 94/365 (25.8%), had poor knowledge, while 271/365 (74.2%) had adequate knowledge. Remarkably, 33/365 (9.0%) thought that their CBHI covered only care from public health institutions. Likewise, 20/365 (5.5%) of the respondents thought that CBHI could cover their expenses outside the country and that it could also cover treatments that were cosmetic in nature. Much as CBHI is a good way of assisting clients in handling their health expenditures, 8/365 (2.2%) of respondents disagreed, while 13/365 (3.6%) didn't know that the scheme doesn't cover their transportation expenses to and from healthcare facilities. The rest of the details on the knowledge of respondents about the CBHI packages are shown in Table 3.

**Table 3.** Respondents' Knowledge on Community Based Health Insurance Scheme Benefit Packages in Central Uganda

Variables	Frequency (N = 365)	Percentage (%)
CBHI is good way of helping clients to health expenditure		
Yes	357	97.8
No	8	2.2
CBHI covers only care from public health institutions		
Yes	33	9.0
No	332	91.0
CBHI covers only care with in the country		
Yes	345	94.5
No	20	5.5
CBHI doesn't cover transportation fee		
Yes	352	96.4
No	13	3.6
CBHI covers only outpatient care		
Yes	353	96.7
No	12	3.3
CBHI also covers inpatient care		
Yes	364	99.7
No	1	0.3
CBHI doesn't cover medical care for cosmetic values		
Yes	345	94.5
No	20	5.5
Answered more than five of the CBHI benefit package correctly		
Yes	271	74.3
No	94	25.7

### Satisfaction With Key Healthcare Services

When the respondents were probed for their satisfaction with the key healthcare services that they had obtained under the CBHI, the majority were generally satisfied. However, there was a considerable group that was undecided as to whether they were satisfied or not. The outstanding aspect of the healthcare service delivery was the insurance co-payment plan, with which 57/365 (15.6%) were unsatisfied. Additionally, ten respondents (2.7%) found the laboratory services in the healthcare facilities inadequate, while undecided respondents towards immunisations, antenatal care, and referral systems were at 34/365 (9.3%), 26/365 (7.1%) and 24/365 (6.6%), respectively. There is a need to highlight the indecisiveness of this category, as they require different strategies compared with the dissatisfied clients when it comes to delivering services that will satisfy them.

### Factors Associated With CBHI Clients' Satisfaction

Overall, the median score for the respondents' satisfaction was 34.0 (IQR: 5.0). Based on this definition, 223/365 (61.1%) of the respondents scored below the median and thus were “not satisfied.” The rest, 142 (38.9%), were above the median and taken to be “satisfied.”

With regards to age, most of those who were unsatisfied and satisfied with CBHI services were individuals who were aged 46 years and above. However, overall, satisfaction did not vary by age (*P*=0.162). Satisfaction decreased with increasing levels of the highest education attained by the respondents. Satisfaction levels were statistically different across the several levels of education (*P*=0.014). Likewise, proportions of those satisfied reduced with increasing distance from the healthcare facilities to the residences of the respondents. There were also statistically significant differences in satisfaction according to the different levels of distance from the facility (p = 0.035). The details on the levels of satisfaction with CBHI services are shown in Table 4.

**Table 4.** Factors Related to Satisfaction with Community Based Health Insurance Scheme Services

Variables	Not satisfied n (%), N = 223	Satisfied n (%), N = 142	P value (Chi-square test)
Age			
<25	28 (12.6)	16 (11.3)	
26-35	57 (25.6)	26 (18.3)	
36-45	43 (19.3)	40 (28.2)	
≥46	95 (42.6)	60 (42.3)	0.162
Sex			
Male	86 (38.6)	56 (39.4)	
Female	137 (61.4)	86 (60.6)	0.868
Education			
No formal education	43 (19.3)	33 (23.2)	
Primary	99 (44.4)	68 (47.9)	
Secondary	75 (33.6)	29 (20.4)	
Certificate and above	6 (2.7)	12 (8.5)	0.014*
Religion			
Christian	190 (85.2)	110 (77.5)	
Moslem	33 (14.8)	32 (22.5)	0.101
Marital status			
Single never married	17 (7.6)	9 (6.3)	
Single divorced / widowed	49 (21.9)	24 (16.9)	
Married	157 (70.5)	109 (76.8)	0.389
Household size			
≤4	30 (13.5)	12 (8.5)	
5 - 8	134 (60.1)	89 (62.7)	
≥9	59 (26.5)	41 (28.9)	0.339
Employment			
Yes	157 (70.4)	100 (70.4)	
No	66 (29.6)	42 (29.5)	0.997
Socioeconomic status			
Lower tertile	133 (59.6)	80 (56.3)	
Middle tertile	55 (24.7)	43 (30.3)	
Highest tertile	35 (15.7)	19 (13.4)	0.473
Health facility level			
Hospital	143 (64.1)	64 (45.1)	
Level IV	9 (4.1)	17 (11.9)	
Level III	58 (26.0)	46 (32.4)	
Private clinic	13 (5.8)	15 (10.6)	0.001
Distance from facility			
≤5km	109 (48.9)	52 (36.6)	
6-10km	70 (31.4)	48 (33.8)	
≥11 km	44 (19.7)	42 (29.6)	0.035
Years on the scheme			
≤ 2	32 (14.4)	39 (27.4)	
3-5	88 (39.5)	44 (31.0)	
≥6	103 (46.2)	59 (41.6)	0.007
Knowledge			
Poor knowledge	45 (20.2)	49 (34.5)	
Good knowledge	178 (79.8)	93 (65.5)	0.002

In the adjusted analysis, those with secondary education were 45% more likely to be less satisfied with CBHI services compared to those with no formal education (adjPR=0.55, 95% CI: 0.36–0.85, *P*=.007). Similarly, being of the Islamic faith was associated with 35% more chances of being satisfied compared with Christians (adjPR=1.35, 95% CI: 1.01–1.79, *P*=.041). Additionally, those who lived further away from the healthcare facilities were 32% less likely to be satisfied with CBHI services compared to those who stayed nearer, i.e., less than five kilometers away (adjPR =0.68, 95% CI: 0.41–0.95, *P*=.014). Those who had been on the scheme for over six years had 32% chances of being unsatisfied compared to those who had been clients for one year or less than one year (adjPR=0.71, 95% CI: 0.51–0.99, *P*=.046). Similarly, there are 24% more chances that a person with good knowledge of the scheme was not satisfied compared to those who had poor knowledge (adjPR=0.76, 95% CI: 0.58–0.99, *P*=.040). The rest of the details are shown in Table 5.

**Table 5.** Multivariable Regression Analysis of Factors Related to Client Satisfaction With Community Based Health Insurance Schemes in Central Uganda

Variables	Crude PR (95%CI)	p-value	Adjusted PR (95% CI)	p-value
Age				
<25	Ref			
26-35	0.86 (0.52 – 1.43)	0.563		
36-45	1.33 (0.85 – 2.08)	0.221		
≥46	1.07 (0.69 – 1.65)	0.780		
Sex				
Male	Ref			
Female	0.98 (0.75 – 1.27)	0.868		
Education				
No formal education	Ref		Ref	
Primary	0.94 (0.68 – 1.29)	0.690	0.86 (0.61 – 1.21)	0.390
Secondary	0.64 (0.43 – 0.96)	0.031	0.55 (0.36 – 0.85)	0.007
Post-secondary	1.49 (0.96 – 2.30)	0.073	1.23 (0.77 – 2.11)	0.344
Religion				
Christian	Ref	Ref		
Muslim	1.36 (1.02 – 1.82)	0.034	1.35 (1.01 – 1.79)	0.041
Marital status				
Single never married	Ref	Ref		
Single divorced / widowed	0.95 (0.51 – 1.78)	0.871	0.86 (0.43 – 1.72)	0.680
Married	1.17 (0.67 – 2.02)	0.181	1.07 (0.57 – 2.05	0.819
Household size				
≤4	Ref	Ref		
5 - 8	1.39 (0.84 – 2.32)	0.195	1.43 (0.81 – 2.51)	0.214
≥9	1.44 (0.84 – 2.45)	0.185	1.55 (0.94 – 2.84)	0.160
Employment				
No	Ref			
Yes	1.01 (0.76 – 1.33)	0.997		
Socioeconomic status				
Lower tertile	Ref		Ref	
Middle tertile	1.17 (0.88 – 1.55)	0.282	1.24 (0.93 – 1.65)	0.141
Highest tertile	0.94 (0.63 – 1.40)	0.194	0.95 (0.64 – 1.46)	0.869
Distance from facility				
≤5km	Ref		Ref	
6-10km	1.26 (0.92 – 1.72)	0.148	1.15 (0.85 – 1.58)	0.364
11-15km	0.9 (0.7 – 2.06)	0.193	0.88 (0.75 – 1.04)	0.142
≥16	0.51 (0.40 – 0.96)	0.009	0.68 (0.41 – 0.95)	0.014
Years on the scheme				
≤ 2	Ref		Ref	
≥3	0.61 (0.44 – 0.84)	0.002	0.71 (0.51 – 0.99)	0.046
Knowledge				
Poor knowledge	Ref		Ref	
Good knowledge	0.66 (0.51 – 0.85)	0.001	0.76 (0.58 – 0.99)	0.040

Additional insights into issues surrounding the satisfaction of respondents with CBHI services were sought from the healthcare providers and the managers of the CBHI. These were synthesized and grouped into themes, as shown in Table 6.

**Table 6.** Provider Perspectives About Client Satisfaction With Community Based Health Insurance Scheme Services in Central Uganda

Themes	Emerging issues
Aspects of patient management at the facility	Waiting time. Absenteeism by the healthcare workers.
Client-related causes of dissatisfaction	Low knowledge of CBHI on insurance packages. Expiry of insurance but client is unaware. Reduction in incomes of clients.
Healthcare facility causes of dissatisfaction	Stock outs of some of the products. High co-sharing costs. Failure to offer subscribed services.
CBHI operational causes of dissatisfaction	Withdrawal of CBHI insurance cards. Longer periods between subscription and commencement of usage.

### Aspects of Patient Management At the Facility

When the healthcare providers were asked about the aspects that the CBHI clients might not have been happy with, they identified the length of time that is taken to verify the clients' documents before they receive medical care. This also fed into the total facility waiting time, hence making the latter generally longer than for the non-CBHI scheme patients. The longer wait times are usually negatively associated with patient satisfaction scores as well as the quality of clinical services. The effect of this is explained by one of the providers below:

*Honestly, when the insurance people come, we are busy at times. And you know we are few. You have to first go to the insurance desk to register the patient and check their documents. But also during that time, there are patients waiting for you to attend to them. Sometimes we take long to serve the insurance people because of that and that at times annoys them. We have had that situation for a long time now, but unless the problem of not enough workers is solved, we cannot do much*.

– Nurse, Health Center II

Additionally, service providers also identified the scarcity and absence of healthcare workers at the facilities as one of the causes of client dissatisfaction. They explain that this was worse during the COVID-19 lockdown, when people kept on reporting to health facilities to seek medical services, but not all healthcare workers could access the facilities.

*During the second lockdown* [June 2021 – August 2021, inclusive] - *in fact, even the first one* [March 2020 – September 2021, inclusive] - *we were locked at home. Even when you wanted to come to the hospital, there was no transport, and the boda-boda* [passenger-carrying motorcycles] *were very expensive. However, patients continued coming and not finding the health workers. We receive many insurance clients here, and they are not happy when they come to get the services that they paid for long ago, but the people are not around. I will not be shocked that they will report to you that they find our services lacking*.

– Medical Officer, Health Center IV

### Client Related Causes of Dissatisfaction

When asked to explain the varying levels of satisfaction, the healthcare workers identified issues such as clients demanding services that are not covered by the CBHI scheme. When patients show up confident that they can access such services or packages, their expectations are dashed when they are told otherwise. The disappointment breeds frustration and dissatisfaction, as one healthcare worker explains:

*By the way, many people come with insurance and demand services. When you check their register, their cards don't allow them to have the services that they are demanding. Some of them remember, but others will show you that they did not know about it. But you can also understand it from this point of view: the person is sick and badly off. Do they even remember which packages they get tor not? For them, they know one thing: they have insurance*.

– Clinical officer, Health Center III

*…….there are other incidences where the person gets signs and symptoms and comes in, for example, thinking they have malaria. The insurance covers them for those simpler diseases. However, when the doctor checks that person, he may find that the case is more serious than what the patient though. Now that case may fall outside the patient's insurance package. That's where trouble starts. The client may not even have the money to pay for anything*.

– Nurse, Hospital

Healthcare providers also explained that at times, clients turned up with expired subscription yet they were not aware. The lack of tracking of expiry of subscription was more common among those clients that stay in rural areas. However, not knowing the expiry date also creates an expectation of access to services, and when the records show ineligibility, the client is dissatisfied with both the healthcare and CBHI services, as one provider explains:

*……. my friend, they come with expired insurance at times. On this, I think the scheme managers are not doing a good job. These people need to be reminded that their cards [*subscriptions*] are about to expire. Otherwise, many of them quarrel as if it were us who made them expire. At least those from the trading centers are okay, but those from villages are the worse ones when it comes to this habit*.

– Clinical Officer, Health Center III

The CBHI managers also explained that clients may be dissatisfied by the fluctuations in incomes. This causes them to leave bigger packages for smaller ones, and this change may frustrate them, especially when they are used to the previous. The respondents explained that the COVID-19 pandemic season made this event very common among the clients, which may explain the dissatisfaction:

*I can even explain this scenario to you. Some people start off well, but when their income reduces - and this can be for any reason, like retrenchment or businesses not doing well during the COVID-19 season such people will struggle to pay for their packages and will bring money in installments. At times, they change packages and go to the basic ones. Now tell me, how will they be satisfied if that happens?*

– CBHI officer

### Healthcare Facility Causes of Dissatisfaction

Much as some clients may have pre-paid for services, there are times when the healthcare facilities cannot serve them due to stock outs. At times, the affected client may not be able to go to another facility because of distance or lack of funds. So this may cause them to be dissatisfied with the CBHI, as one provider explains:

*…..when some of our drugs reduce or we run out of stock, we cannot serve the patients. And this is at times common. How do you expect a person who paid their money to be served to behave if they are not served? Of course they will be annoyed. For me it would even be worse…..*

– Nurse, Hospital

Due to fluctuating market prices of hospital inputs, there are times when the pricing of services is adjusted. When it goes higher, it means that the contribution by the client due to cost-sharing will also go up. This affects the CBHI clients when they are told that, despite the co-sharing percentage remaining constant, the cost has increased. It was identified as one of the sources of dissatisfaction:

*Sometimes, hospital requirements go up on the open market, meaning that the money they have to top up for treatment also goes up. These definitely affect the way they perceive us*.

– CBHI manager

### CBHI Operational Causes of Dissatisfaction

The CBHI also has operational challenges that are potential sources of dissatisfaction. When asked about the periods between subscription and commencement of usage, CBHI managers explained that some clients want to start consuming services immediately, yet the policy states otherwise. This means that during this period, the clients will pay out-of-pocket (OOP) upon seeking services at healthcare facilities. At times, this is not taken in good spirit. However, the managers also explained that there are times they get an upsurge of claims without justifiable cause. The intervention they instituted was to withdraw the CBHI cards from clients. This resulted in an uproar, but the managers insisted it was the right thing to do at that time:

*There are those who just want to pay and bring their family to the hospital the following week, despite being told the terms at the beginning. When they are told that the insurance will not be able to pay for that, they shout at us. By the way, during the first lockdown, claims rose. ……we are sure it was not COVID, but the claims somehow rose. We withdrew cards to slow down the rate. Each hour I would receive a phone call with someone quarreling*.

– CBHI officer

## DISCUSSION

The level of satisfaction with CBHI services in the study area was 38.9%. The factors that were negatively associated with satisfaction to CBHI services included having secondary as the highest level of education; residing beyond 16 kilometers away from the healthcare facility; having been on the CBHI scheme for a longer time; and having good knowledge about the CBHI. In addition, with being of Islamic faith having higher chances of being satisfied compared with Christians. Healthcare providers identified impediments to client satisfaction as: their irregular presence during the COVID-19 lockdown period; taking too long to verify client details; clients demanding for services that are not covered by the CBHI scheme; clients turning up with expired subscription; and failing to provide services. From the perspective of CBHI scheme managers, issues affecting client satisfaction included lag time after paying the premium; decision to withdraw insurance during the COVID-19 period; and reduction in incomes of clients.

This study found that there were more females than males that sought care at the healthcare facilities which were selected to be part of the research. This may be explained by the gender differences in healthcare seeking behavior. The findings are in agreement with earlier studies that pointed out that women are better at seeking medical care. In Canada, women reported that they had visited their primary care provider to a greater extent than did men.^22^ Similarly in South Africa, research reported findings in the same direction.^23^ Still, in this study, there were more people of older age reporting for healthcare. It should be noted that age has been found as a key risk factor for various diseases.^24^ Therefore, this finding in our study is in agreement with the broader scientific base.

The commonest reason for enrolling was the respondent having heard about the good experiences of those that were on the CBHI schemes. The social networks that transmit information about community programs on health cannot be discounted. In their earlier study about the role of social networks and information sharing in Uganda, Nshakira-Rukundo et al, found that sharing information and better social networks were positively associated with enrolment in community health insurance schemes.^25^ This means that the finding in this study that many respondents joined because they had heard of the benefits from others is not uncommon in the Ugandan setting.

In this study 25% of the CBHI clients had poor knowledge of the scheme and its services. In the African region, there has been inadequate literature that explains insurance clients' satisfaction anchored on knowledge and awareness of enrollees. In fact, one study in West African called for such studies to be initiated.^26^ Indeed some studies in the low income countries have found that satisfaction with health insurance schemes is dependent on the enrollee's knowledge of the benefit packages.^11,27^ This underscores the importance of our study having examined this variable. However, our findings contrast those in similar settings which reported prevalence of good knowledge among CBHI clients to be 20% higher than in our study.^10^

In this study, the level of satisfaction with the CBHI schemes was established to be 38.9%. These results are comparable to two studies that were earlier conducted in Nigeria which found the satisfaction to be 42% and 46%.^11,28^ Markedly, our finding is far lower than the household satisfaction of 91.38 % which was reported in one of the studies in Ethiopia.^5^ The differences here may be explained by the approaches that were used to define *‘satisfaction’*. Whereas our study used a median score as a cut-off point, in that comparative study in Ethiopia, the scores of satisfaction were calculated based on the percentage of maximum scores. This approach has the full potential of overestimating the proportion of those that are satisfied with the CBHI. Another explanation to this difference may be that the location of the comparative study is completely different from our study and the very high level of satisfaction may be related to greater and better levels of the quality of health services given under the insurance. From the methods approach, the comparative study measured the overall satisfaction score by asking only six questions. In our study, we based satisfaction on seven questions which might have resulted into probing more problems and challenges related to the CBHI. This said, the proportion of those satisfied with CBHI in our study was slightly lower than what different studies conducted in Istanbul city, Turkey^29^ as well as Nigeria^30^ reported.

When it comes to the relationship between socio-demographic characteristics and health insurance satisfaction, a number of studies have been conducted. Some of these studies have shown than health insurance clients' satisfaction is associated with socio-demographic characteristics like marital status, age, gender/sex, family size, and education status.^5,11,29^ In our study, there were differences between those that were satisfied and those that were not, based on the highest levels of education attained. Specifically, this is in agreement with one of the earlier studies mentioned.^29^

Still in this study, most of the socio-demographic characteristics were not statistically significantly associated with satisfaction levels after controlling for potential confounders. This is perhaps not a strange finding because similar result have been published in India. In the comparative study, socio-demographic and economic covariates such as age, gender, education/literacy level and economic status were similarly found not be significantly associated with the contentment of clients of a health insurance scheme.^31^

Nonetheless, in our study, modifiable factors such as knowledge of the CBHI benefit packages were significantly associated with satisfaction. It is expected that the clients' knowledge of the CBHI scheme was a key determinant of their perceived satisfaction. We specifically included this variable because of the understanding that good knowledge was one of the ways of promoting satisfaction. This was extracted from the earlier studies that concluded that poor knowledge of the benefit package is negatively related to the rates of utilization of health facilities in low-income countries.^27^

Much as it was expected that poor knowledge of health insurance results into to less contentment with health service provision, this study found that good knowledge of the scheme actually led to less satisfaction. This may be explained by the fact that the more enrollees knew about the schemes, then the more they were likely to discover the gaps. In Nigeria, there are reports that those with sufficient knowledge always raise complaints for example when they are denied their full entitlements.^32^ However, our findings are in contrast with those of a study in Nigeria which found better knowledge to be associated with better satisfaction.^11^ The same explanation may apply to the similar observations for higher levels of education and having stayed on the scheme for a longer time.

In this study, the further one stayed away from the healthcare facility, the more chances of being less satisfied with the CBHI scheme. This is not a peculiar finding because earlier studies have pointed out that high transport costs to and from the hospitals mask the visible advantages of scheme membership.^6^ In addition, in a systematic review on the barriers and facilitators to implementation, acceptance / uptake and sustainability of CBHI schemes in low- and middle-income countries, it has been realized before that large distances to in-network health facilities formed a huge obstacle to enrolment. Further, to a large extent, distance has also been cited as a reason for non-renewal of membership into CBHI schemes.^33^

We found that those of the Islamic faith having a higher likelihood of satisfaction compared to Christians. Studies have revealed that patient satisfaction can be affected by a variety of factors, including a patient's individual characteristics like religion. That our study found religion of the client to be a significant predictor of satisfaction is in agreement with some studies. When a secondary data analysis was conducted in USA, it was demonstrated that patients who believed that religion was a very important part of their life showed higher levels of patient satisfaction.^34^ However, this contradicts the findings of a systematic review that revealed that there was a little evidence of whether religion affected overall patient satisfaction.^35^ Nonetheless, the differences in levels of satisfaction by religion, for example between Christians and Moslems would constitute a key area for further academic exploration.

One of the issues identified by healthcare providers as affecting client satisfaction was the irregular presence of providers during the COVID-19 lockdown period. This concurs with earlier studies that have elaborately explained how absenteeism of health workers in healthcare facilities is widespread in low-income countries.^36^ Indeed, other authors have also found linkages between this absenteeism and poor patient satisfaction.^37^ Additionally, there is a likelihood that taking too long to verify client details affected client satisfaction through the pathway of increased waiting time. A number of studies have demonstrated that prolonged waiting times result in low patient satisfaction.^38,39^ Similarly, anything that denies a service to a patient will cause dissatisfaction and this explains why clients demanding for services that are not covered by the CBHI scheme; expired subscription; failing to provide services; and the intervention of withdrawing of insurance cards during the COVID-19 period were associated with reduced satisfaction.

We conclude that in the study area, the level of satisfaction with CBHI services was considerably low despite the schemes having existed for more than two decades. Additionally, clients who were better educated, lived far from the healthcare facilities that serve them, had stayed longer on the scheme and had good knowledge of the schemes were likely to be less satisfied. Lockdowns due to pandemics interrupt healthcare services and subsequently affect the satisfaction of CBHI clients with scheme services. We recommend that CBHI managers identify healthcare facilities that are nearer to the clients. In addition, scheme and healthcare facility managers should also invest in client management and processing technologies in order to cut down the waiting time. Further, government should design disease control interventions that do not interrupt healthcare service delivery.

### Study Limitations

The study was conducted in selected healthcare facilities in central Uganda, which may not be representative of all CBHI schemes or regions in Uganda. This limits the generalisability of the findings to other settings.

The cross-sectional nature of the study captures client satisfaction at a single point in time, making it difficult to assess changes in satisfaction over time or determine causality between the identified factors and satisfaction levels.

## CONCLUSION

The study revealed that client satisfaction with Community-Based Health Insurance (CBHI) services during the COVID-19 pandemic was relatively low, with only 38.9% of participants expressing satisfaction. Factors contributing to lower satisfaction included higher levels of education, longer distances from healthcare facilities, longer duration of scheme membership, and better knowledge of CBHI benefits. The irregular availability of healthcare workers and long waiting times were also significant issues affecting client satisfaction.

These findings underscore the need for scheme managers to address these challenges by identifying facilities closer to enrollees and investing in technologies to reduce waiting times. Enhancing the availability of healthcare workers and improving communication about the benefits and operations of CBHI schemes can also help improve client satisfaction. Ensuring that CBHI schemes are resilient during pandemics and other disruptions is crucial for maintaining and improving client satisfaction and ultimately achieving universal health coverage.
